# Phytochemical Analysis and Antimicrobial Activities of Methanolic Extracts of Leaf, Stem and Root from Different Varieties of *Labisa pumila* Benth

**DOI:** 10.3390/molecules16064438

**Published:** 2011-05-27

**Authors:** Ehsan Karimi, Hawa Z.E. Jaafar, Sahida Ahmad

**Affiliations:** 1 of Crop Science, Faculty of Agriculture, Universiti Putra Malaysia, Serdang, Selangor 43400, Malaysia; 2 Department of Biochemistry, Faculty of Biotechnology and Biomolecular Sciences, Universiti Putra Malaysia, Serdang, Selangor 43400, Malaysia

**Keywords:** flavonoids and phenolics, saponin content, HPLC analysis, antibacterial activity, antifungal activity

## Abstract

A local herb, Kacip Fatimah, is famous amongst Malay women for its uses in parturition; however, its phytochemical contents have not been fully documented. Therefore, a study was performed to evaluate the phenolics, flavonoids, and total saponin contents, and antibacterial and antifungal properties of the leaf, stem and root of three varieties of *Labisia pumila* Benth. Total saponins were found to be higher in the leaves of all three varieties, compared to the roots and stems. Leaves of var. *pumila* exhibited significantly higher total saponin content than var. *alata* and *lanceolata*, with values of 56.4, 43.6 and 42.3 mg diosgenin equivalent/g dry weight, respectively. HPLC analyses of phenolics and flavonoids in all three varieties revealed the presence of gallic acid, caffeic acid, rutin, and myricetin in all plant parts. Higher levels of flavonoids (rutin, quercitin, kaempferol) were observed in var. *pumila* compared with *alata* and *lanceolata*, whereas higher accumulation of phenolics (gallic acid, pyrogallol) was recorded in var. *alata*, followed by *pumila* and *lanceolata.* Antibacterial activities of leaf, stem and root extracts of all varieties determined against both Gram positive (*Micrococcus luteus*, *Bacillus subtilis* B145, *Bacillus cereus* B43, *Staphylococcus aureus* S1431) and Gram negative (*Enterobacter aerogenes*, *Klebsiella pneumonia* K36, *Escherichia coli* E256, *Pseudomonas aeruginosa* PI96) pathogens showed that crude methanolic extracts are active against these bacteria at low concentrations, albeit with lower antibacterial activity compared to kanamycin used as the control. Antifungal activity of methanolic extracts of all plant parts against *Fusarium* sp., *Candida* sp. and *Mucor* using the agar diffusion disc exhibited moderate to appreciable antifungal activities compared to streptomycin used as positive control.

## 1. Introduction

As a natural resource plants have a high potential for producing new drugs of great benefit to humans. There are many approaches to looking for new biologically active values in plants [1]. The diseases that have been managed successfully by using herbal medicine include malaria, epilepsy, infertility, convulsion, diarrhea, dysentery, gonorrhea, flatulence, tonsillitis, bacterial and fungal infections, mental illness and worm infections [2]. Natural selection during evolution and competition between organisms has biologically produced powerful and active natural products which can serve as leads and have been refined by modern techniques in order to give more specifically active drugs [3]. The most important of these bioactive constituents, which are mainly secondary metabolites, are alkaloids, saponin, flavonoids, tannins and phenolic compounds. These phytochemicals could be toxic to microbial cells [4]. 

There has been intense interest in plant polyphenols as witnessed by the numerous papers devoted to various aspects of these compounds [5,6]. Polyphenols are plant metabolites characterized by the presence of several phenol groups (*i.e.*, aromatic rings with hydroxyls), which derive from L-phenylalanine [7]. The most important classes of polyphenols are phenolic acids, which include polymeric structures, such as hydrolyzable tannins, lignans, stilbenes, and flavonoids. Flavonoids include flavonols, flavones, isoflavones, flavanones, anthocyanidins among which flavanols are most commonly known for their antioxidant and antimicrobial biological activities [8,9].

Saponins are a diverse group of phytochemicals whose chemical structures are composed of a fat-soluble nucleus (aglycone) which is either a triterpenoid (C-30) or a neutral or alkaloid steroid (C-27) attached to one or more water-soluble sugars (glycone) side chains through ester linkages to the aglycone nucleus at different carbon sites [10]. Triterpenoid saponins predominate in soybean, alfalfa and quillaja [10], whereas steroid saponins are predominant in yucca, tomato and oats [10]. The various biological effects of saponins include haemolytic and antibacterial activities [11].

*Labisia pumila* (Myrsinaceae), also locally known as Kacip Fatimah (KF), has been used by many generations to induce and facilitate childbirth as well as a post-partum medicine by Malay women [12]. There are three varieties of KF in Malaysia, namely, *Labisia pumila* var. *alata*, *L. pumila* var. *pumila* and *L. pumila* var. *lanceolata* [13]. Recently, researches have demonstrated KF estrogenic activity and high amount of phenolics in this sp. [14]. Hence, *L. pumila* contains chemicals including alkenyl resorcinol, flavonoid and benzoquinones [15]. There is also a report on the presence of alkyl and alkenyl resorcinols in *alata* and *pumila* varieties which cause irritation, inflammation and blistering of the skin [16]. Therefore, the aims of the present research were to analyze the flavonoids and phenolics and total saponin content, and to evaluate the antimicrobial activities of the leaves, stems, and roots of three varieties of *Labisia pumila* Benth.

## 2. Results and Discussion

### 2.1. Analyses of Phenolic and Flavonoid Compounds by RP-HPLC

In this study reversed-phase (RP) chromatography was used to determine the flavonoid and phenolic compounds in methanolic leaf, stem and root extracts of three varieties of *Labisia pumila*. In all three varieties, the leaf part contained higher levels of phenolic and flavonoid compounds compared to root and stem. The results obtained from the HPLC analysis of flavonoids are listed in Table 1. It was shown that kaempferol, naringin and myricetin were the main flavonoids in all three varieties with respective values of 222.8, 79.4, 31.1 µg/g dry sample in the leaves of var. *pumila*, 187.2, 140, 88.4 µg/g dry sample of var. *alata*, and 164.6, 86.7, 27.9 µg/g dry sample of var. *lanceolata*. Quercitin and rutin were only recorded in var. *pumila* (106.7 and 24.1 µg/g dry sample, respectively) and *lanceolata* (57.1 and 19.43 µg/g dry sample, respectively).

**Table 1:** Concentration of different flavonoid compounds from methanolic extract of leaf, stem and root of three varieties of *Labisia pumila* Benth.

Sample	Flavonoid contents (µg/g dry sample)				
	Kaempferol	Myricetin	Naringin	Quercetin	Rutin
*Alata* Leaf	187.2 ± 0.03 ^b^	88.4 ± 0.06 ^a^	140 ± 0.01 ^a^	ND	ND
*Alata* Root	19.7 ± 0.08 ^d^	19.3 ± 0.13 ^d^	ND	ND	ND
*Alata* Stem	11.7 ± 0.23 ^e^	ND	21.6 ± 0.23 ^d^	ND	4.6 ± 0.03 ^d^
*Pumila* Leaf	222.8 ± 0.05 ^a^	31.1 ± 0.013 ^b^	79.4 ± 0.03 ^c^	106.7 ± 0.05 ^a^	24.1 ± 0.023 ^a^
*Pumila* Root	20.7 ± 0.46 ^d^	ND	ND	50.4 ± 0.03 ^c^	5.2 ± 0.04 ^c^
*Pumila* Stem	ND	ND	ND	48.5 ± 0.007 ^d^	19.4 ± 0.01 ^b^
*Lanceolata* Leaf	164.6 ± 0.03 ^c^	27.9 ± 0.04 ^c^	86.7 ± 0.43 ^b^	57.1 ± 0.02 ^b^	ND
*Lanceolata* Root	ND	ND	ND	ND	ND
*Lanceolata* Stem	ND	ND	ND	ND	25.1 ± 0.025 ^a^

ND: not detected. All analyses were mean of triplicate measurements ± standard deviation. Means not sharing a common letter were significantly different at p ≤ 0.05.

Overall, higher flavonoids were observed in *Labisia pumila* var. *pumila* compared to var. *alata* and var. *lanceolata*. The analyses also confirmed the presence of gallic acid and caffeic acid as the main phenolics compounds in all three varieties. However, pyrogallol was only observed in *Labisia pumila* var. *alata* (811.2 µg/g dry sample) (Table 2). As three varieties were being compared with each other, leaf of var. *alata* exhibited higher total accumulation of gallic acid (449. 53 µg/g dry sample) followed by var. *pumila* (216.42 µg/g dry sample) and var. *lanceolata* (407.84 µg/g dry sample). These values were higher than the gallic acid value which had been previously found in fresh Mauritian black tea leaves (0.006 µg/g dry weight) [17], and three other medicinal plant species (*i.e.*, *Tectona grandis*, *Shilajit* spp., and *Valeriana wallachi*) with concentrations of 7.80, 20.76 and 17.48 µg/g dry weight respectively [18]. 

**Table 2:** Concentration of different phenolic compounds from methanolic extract of leaf, stem and root of three varieties of *Labisia pumila* Benth.

Sample	Phenolic contents (µg/g dry sample)		
	Gallic acid	Pyrogallol	Caffeic acid
*Alata* Leaf	449.5 ± 0.003 ^a^	811.2 ± 0.05 ^a^	48.9 ± 0.03 ^c^
*Alata* Root	145.5 ± 0.01 ^d^	343.2 ± 0.03 ^b^	ND
*Alata* Stem	19.1 ± 0.15 ^h^	111.4 ± 0.02 ^c^	ND
*Pumila* Leaf	216.4 ± 0.04 ^c^	ND	44.4 ± 0.13 ^d^
*Pumila* Root	37.8 ± 0.03 ^f^	ND	24.5 ± 0.03 ^e^
*Pumila* Stem	18.1 ± 0.35 ^h^	ND	69.6 ± 0.025 ^b^
*Lanceolata* Leaf	407.8 ± 0.03 ^b^	ND	116.1 ± 0.04 ^a^
*Lanceolata* Root	36.6 ± 0.01 ^g^	ND	ND
*Lanceolata* Stem	63.4 ± 0.03 ^e^	ND	ND

ND: not detected. All analyses were mean of triplicate measurements ± standard deviation. Means not sharing a common letter were significantly different at p ≤ 0.05.

**Figure 1:**
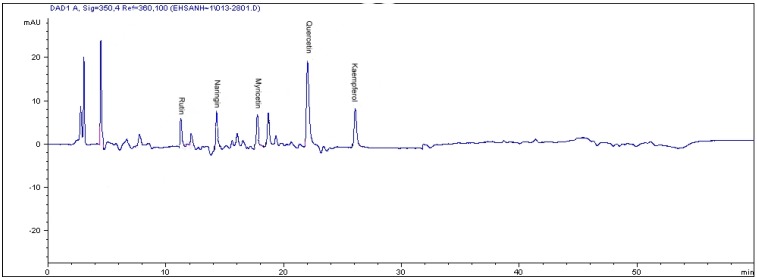
The RP-HPLC chromatogram of flavonoid compounds in the leaves of *Labisia pumila* var. *pumila.* Identification of compounds: rutin, naringin, myricetin, quercetin and kaempferol.

**Figure 2:**
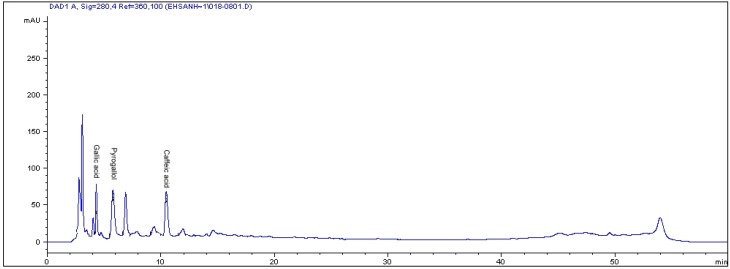
The RP-HPLC chromatogram of phenolic compounds in the leaves of *Labisia pumila* var. *alata.* Identification of compounds: gallic acid, pyrogallol and caffeic acid.

The pyrogallol contents detected in this study for leaf, root and stem of *L. pumila* var*. alata* (811.2, 343.2 and 111.4 µg/g dry weight, respectively) were lower than those documented in seagrass *Posidonia aceanica* (1.3 mg/g dry weight) [19]. On the contrary, in this study kaempferol contents in the leaves of the three varieties of *Labisia pumila* were found to be higher than that reported in red wine and *Ginkgo biloba* (6.90 and 15µg/g fresh weight tissue, respectively) [20], but these values were lower when compared to raw propolis and Mauritian black tea leaves (722 and 570 µg/g dried weight tissue, respectively) [17]. Leaf extract of *Labisia pumila* var. *pumila* and *lanceolata* contained lower concentrations of rutin compared with raw gingko leaves, which registered 1,880 μg/g sample as reported by Goh and Barlow [21]. Compared to onion and garlic, leaf extracts of *Labisia pumila* var. *pumila* had higher quercetin content. The concentrations of quercetin in onion and garlic leaves were 24 ± 2.4 and 82 ± 8.6 μg/g dry weight, respectively [22]. The HPLC chromatograms show the flavonoid compounds in the leaves of *Labisia pumila* var. *pumila* (Figure 1) and phenolic compounds in the leaves of *Labisia pumila* var. *alata* (Figure 2) as examples.

### 2.2. Total Saponin Content

Saponins are bioactive compounds with a wide range of medicinal properties, including hypo-cholesterolemic, anticarcinogenic, anti-inflammatory, antimicrobial and antioxidant activities [23]. Saponins are found in a number of plant-derived foods such as legumes, and are present in many medicinal plants, including ginseng [24]. Results on the saponin contents in the leaves, stems and roots of three varieties of *Labisia pumila* showed significant differences (p < 0.05). In all three varieties, the leaf parts contained higher saponin levels than root and stem (Table 3). *Labisia pumila* var. *pumila* had a higher total saponin content in the leaves, stem and root, respectively, at 56.4, 37.3 and 41.2 mg diosgenin equivalent/g DM, compared to those recorded in var. *alata* (43.6, 24.5 and 34.1 mg diosgenin equivalent/g DM, respectively) and var*. lanceolata* (42.3, 20.8 and 33.7 mg diosgenin equivalent/g DM, respectively). Previous researchers have also reported that alfalfa contains 0.5-9.5% [25], licorice root contains more than 3% [26], fenugreek seed contains 5-6% [24] and the aerial parts of *Medicago arborea* contain 1.9-3.4% saponins in dry matter [27]. Moreover, the saponin content in some other crops were guar meal 5-13% [28], soybean seed (*Glycine max*) 0.5-6.5% [29], yucca 8-12% [30], and quillaja 8-10% saponin [31].

**Table 3:** Total saponin content (mg diosgenin equivalent/g DM) in the leaf, stem and root from three varieties of *Labisia pumila.*

Variety	Leaf	Stem	Root
*Alata*	43.6 ± 0.28 ^b^	24.5 ± 0.11 ^f^	34.1 ± 0.13 ^e^
*Pumila*	56.4 ± 0.13 ^a^	37.3 ± 0.25 ^d^	41.2 ± 0.12 ^c^
*Lanceolata*	42.3 ± 0.29 ^c^	20.8 ± 0.82 ^g^	33.7 ± 0.99 ^e^

Values are means of three replications; means with the different letters are significantly different.

### 2.3. Antibacterial Activity Analysis

The antibacterial activity of leaf, stem and root extracts from the three varieties were tested using the disc diffusion method at a concentration of 300 µg/disc. The results, shown in Table 4, indicate significant differencea (p < 0.05) in inhibitory activity between leaf, stem and root in all three varieties of *Labisia pumila* Benth. These extracts exhibited moderate to appreciable antibacterial activities against four Gram-positive and four Gram-negative bacteria. In all three varieties, the leaves had higher activity compared to roots and stems. The *Labisia pumila* var. *pumila* had higher activity against Gram positive bacteria compared to var. *alata* and *lanceolata*, while *Labisia pumila* var. *alata* had a higher activity against Gram negative bacteria compared to var. *pumila* and *lanceolata*. In all the three varieties, the leaf extracts at the concentration of 300 µg/disc showed different inhibitory activity against all the Gram positive bacteria (*Micrococcus luteus*, *Bacillus subtilis*, *Bacillus cereus* and *Staphylococcus aureus*), with inhibition zone diameters ranging from 0.55 to 1.15 cm. At the same time, these extracts showed various inhibitory activities against all the Gram negative bacteria (*Enterobacter aerogenes*, *Klebsiella pneumonia*, *Escherichia coli*, and *Pseudomonas aeruginosa*) with inhibition zone diameters ranging from 0.49 to 1.32 cm. Kanamycin as a standard antibiotic showed high inhibition zones at a concentration of 1 µg/disc.

Flavonoids and phenolic compounds act as antibacterial agent against many pathogenic bacteria such as *Staphylococcus aureus*, *Staphylococcus epidermis*, *Bacillus cereus*, *Bacillus subtilis*, *Pseudomonas aeruginosa*, *Klebsiella pneumonia*, *Salmonella typhi* and *Escherichia coli* [32]. Flavonoids such as chrysin, genistein, luteolin, and quercetin-4’-methyl ether in *Calycotome villosa* were identified as being responsible for the inhibition growth of *Bacillus lentus*, *Escherichia coli*, and *Klebsiella pneumoniae* [33]. Gallic acid isolated from *Rubus ulmifolis* exhibited antimicrobial activity against *Staphylococcus aureus*, *Bacillus cereus*, *Escherichia coli* and *Candida albicans* [34]. Beside phenolics and flavonoids, many saponins have antimicrobial activity that is affected by factors such as the aglycone, number, position and chemical structure of sugar side chains [35]. Cheeke *et al.* [36] indicated that addition of saponin-rich yucca powder or extract to ruminant animals’ diets resulted in more antimicrobial activity against Gram-positive bacteria (*Staphylococcus aureus*) than Gram-negative bacteria (*Escherichia coli*). Some saponins exhibit antimicrobial activity against Gram-positive bacteria and no activity against Gram-negative bacteria in this study when a saponin-rich extract was tested. The same observation was reported by Oleszek *et al*. [26] who had noted that the saponin fraction isolated from soapnut pericarps had showed moderate antibacterial activity against Gram-positive bacteria, while no activity was observed against Gram-negative bacteria. Conversely, saponins isolated from roadside tree (*Bauhinia variegata* L.) bark exhibit greater antibacterial activity for Gram-negative bacteria than Gram-positive bacteria at concentrations ranging from 2.5 to 10 mg/mL [37]. In addition Kuete *et al*. [38] reported significant antibacterial effects of saponin-rich methanolic extracts from the stem bark of African *Tridesmostemon omphalocarpoides*.

**Table 4:** Inhibition zones of leaf, stem and root extracts of three varieties of *Labisia pumila* against pathogenic bacteria at concentration of 300 µg/disc.

Sample*	Inhibition Zone (cm)			
	Gram-positive bacteria			
	*B. subitilis*	*S. aureus*	*B. cereus*	*M. luteus*
*Pumila* Leaf	1.15 ^b^	0.95 ^b^	1.05 ^b^	0.57 ^c^
*Alata* Leaf	1.05 ^c^	0.91 ^bc^	1.1 ^b^	0.65 ^b^
*Lanceolata* Leaf	1.00 ^cd^	0.80 ^c^	0.96 ^c^	0.55 ^c^
*Pumila* Root	0.99 ^cd^	0.65 ^d^	0.96 ^c^	0.41 ^d^
*Alata* Root	0.94 ^ed^	0.39 ^ef^	0.85 ^ef^	0.39 ^de^
*Lanceolata* Root	0.96 ^cde^	0.49 ^e^	0.94 ^cd^	0.36 ^def^
*Pumila* Stem	0.93 ^de^	0.31 ^f^	0.89 ^de^	0.37 ^def^
*Alata* Stem	0.89 ^e^	0.29 ^f^	0.79 ^fg^	0.35 ^ef^
*Lanceolata* Stem	0.79 ^f^	0.33 ^f^	0.73 ^g^	0.33 ^f^
Kanamycin	1.25 ^a^	1.11 ^a^	1.28 ^a^	0.88 ^a^
	**Gram-negative bacteria**			
	***E. coli***	***P. aeruginosa***	***E. aerogenes***	***K. pneumonie***
*Pumila* Leaf	1.26 ^c^	0.53 ^c^	1.12 ^c^	1.12 ^b^
*Alata* Leaf	1.32 ^b^	0.75 ^b^	1.23 ^b^	1.05 ^b^
*Lanceolata* Leaf	1.15 ^d^	0.49 ^cd^	1.05 ^d^	0.95 ^c^
*Pumila* Root	0.93 ^f^	0.47 ^d^	0.98 ^e^	0.62 ^f^
*Alata* Root	1.01 ^e^	0.47 ^d^	1.11 ^cd^	0.83 ^d^
*Lanceolata* Root	0.97 ^ef^	0.33 ^e^	0.96 ^ef^	0.73 ^e^
*Pumila* Stem	0.67 ^g^	0.44 ^d^	0.9 ^f^	0.91 ^c^
*Alata* Stem	0.50 ^h^	0.44 ^d^	0.79 ^g^	0.81 ^de^
*Lanceolata* Stem	0.45 ^h^	0.37 ^e^	0.71 ^h^	0.82 ^d^
Kanamycin	1.48 ^a^	1.03 ^a^	1.33 ^a^	1.31 ^a^

Means with different superscripts within column are significantly different (P < 0.05); * sample of methanolic extract and standard antibiotic agent.

### 2.4. Antifungal Activity Determination

The results of the antifungal activity of leaf, stem and root extracts of three varieties of *Labisia pumila* Benth. are presented in Table 5. The leaves in all three varieties showed higher activity towards the tested fungi as compared to root and stem at a concentration of 500 µg/well. The highest inhibitory activity was observed in the methanolic extract of leaf in variety *alata* against *Fusarium* sp. (inhibition zone = 0.75 cm) and in variety *pumila* with the highest inhibitory activity found against *Candida* sp. (inhibition zone = 0.79 cm) and *Mucor* sp. (inhibition zone = 0.69 cm). Among the different fungi tested, *Candida* sp. was found to be the most sensitive to all the extracts. Although the mechanism of action of such compounds against fungi is still unknown, their efficacy, availability, low cost and low toxicity to humans give the phenolic acids and flavonoids great potential as natural fungicides compounds [39]. According to Cushnie and Lamb, [40] the application of flavonoids against pathogenic fungi has been considered since they were known as spore germination of plant pathogens inhibitors. Davidyants *et al.* [41] evaluated the anti-fungal properties of triterpene saponins from *S. perfoliatum*, where a mixture of the saponins significantly inhibited the growth of *Dhreslera graminea* and inhibited both mycelia growth and spore formation of *Rhizopus nodosus* and *Rhizopus nigricens* [41]. In addition saponins isolated from *Calendula officinalis*, also a member of the *Asteraceae*, exhibited both fungicidal and anti-bacterial activities [42].

**Table 5:** Inhibition zones of leaf, stem and root extracts of three varieties of *Labisia pumila* against pathogenic fungi at concentration of 500 µg/well.

Sample *	Inhibition Zone (diameter in cm)		
	*Fusarium* sp.	*Candida* sp.	*Mucor* sp.
*Pumila* Leaf	0.62 ^c^	0.79 ^b^	0.69 ^b^
*Alata* Leaf	0.75 ^b^	0.70 ^c^	0.63 ^b^
*Lanceolata* Leaf	0.57 ^c^	0.60 ^de^	0.47 ^c^
*Pumila* Root	0.58 ^cd^	0.54 ^ef^	0.45 ^c^
*Alata* Root	0.59 ^cd^	0.65 ^cd^	0.43 ^c^
*Lanceolata* Root	0.41 ^f^	0.45 ^gh^	0.31 ^de^
*Pumila* Stem	0.47 ^ef^	0.50 ^fg^	0.36 ^d^
*Alata* Stem	0.52 ^de^	0.55 ^ef^	0.35 ^d^
*Lanceolata* Stem	0.30 ^g^	0.40 ^h^	0.28 ^e^
Streptomycin	1.41 ^a^	1.43 ^a^	0.90 ^a^

Means with different superscripts within column are significantly different (P < 0.05); * sample of methanolic extract and standard antibiotic agent.

## 3. Experimental

### 3.1. Plant Material

Seedlings of *Labisia pumila* varieties *alata*, *pumila*, and *lanceolata* werecollected from places of origin at Sungkai, Perak; Hulu Langat, Selangor, and Kota Tinggi, Johore, respectively, and raised under similar glasshouse conditions for 18 months before being used in the study. The GPS location details were 3°0'35.27"N latitude and 101°42'19.38"E longitude. Healthy and uniform seedlings in term of leaf numbers were selected from the three varieties. The leaf, stem and root of *Labisa pumila* Benth. were cleaned, separated, and freeze dried for further analysis.

### 3.2. Preparation of Extracts

Samples were extracted using methanol as a solvent as described by Crozier *et al.* [43]. Two grams of freeze-dried leaf, stem and root were weighed and placed into a 100 mL conical flask, and treated with 80% (v/v) aqueous methanol (40 mL). It was followed by an addition of 6 M HCl (10 mL). The mixture was refluxed for 2 hours at 90 °C and filtered using Whatman No. 1 filter paper (Whatman, England) followed by evaporation of the filtrate using a vacuumed Rotary Evaporator (Buchi, Switzerland). The crude extracts were re-dissolved in methanol for antimicrobial, antifungi and RP-HPLC analyses.

### 3.3. Analyses of Phenolic and Flavonoid Compounds by RP-HPLC

The phenolic and flavonoid compounds of the leaf, stem and root of *Labisa pumila* were quantitatively measured by reversed-phase HPLC based on the method described by Crozier *et al.* [43] with some modifications. Phenolic standards used were gallic acid, caffeic acid and pyrogallol. Flavonoid standards were quercetin, rutin, myricetin, kaempferol and naringin at stock concentrations of 100 µg/mL. An aliquot of sample extract was loaded on the HPLC equipped with an Intersil ODS-3 (5 μm 4.6 × 150 mm, Gl Science Inc) analytical column. Solvents comprising deionised water (solvent A) and acetonitrile (solvent B) were used. The pH of water was adjusted to 2.5 with trifluoroacetic acid. The phenolic and isoflavonoid compounds were detected at 280 nm while flavonoid compounds at 350 nm. The column was equilibrated by 85% solvent A and 15% solvent B. Then the ratio of solvent A was increased to 85% in 50 min followed by reducing solvent B to 15% in 55 min. This ratio was maintained to 60 min for the next analysis with flow rate at 0.6 mL/min.

### 3.4. Total Saponin Content

Total saponin content was determined according to Makkar and Becker [44] based on the vanillin-sulfuric acid colorimetric reaction. The results were expressed as mg diosgenin equivalent per gram dry matter of the plant material.

### 3.5. Antibacterial Activity Assay

The antibacterial assay of leaf, stem and root of *Labisia pumila* extracts was carried out against *Staphylococcus aureus* S1431, *Escherichia coli* E256, *Pseudomonas aeruginosa* PI96, *Micrococcus luteus*, *Klibsiella pneumonia* K36, *Bacillus subtilis* B145, *Bacillus cereus* B43 and *Enterococcus aerogenes* by the disc diffusion method as described by Boussaada *et al*. [45]. All the bacteria were purchased from the Institute of Malaysian Research (IMR) and maintained in the Department of Microbiology, Faculty of Biotechnology and Bimolecular Sciences, Universiti Putra Malaysia. In this assay, the positive control without extracts (solvent) and reference control used kanamycin as the standard antibiotic agent. The extracts inhibitions were corrected based on positive control values and compared to those of reference control. The experiments were run in triplicate. 

### 3.6. Antifungi Activity Assay

The antifungal assay was carried out by the agar well diffusion assay [46] with slight modifications. Briefly, a suspension of the tested fungi was prepared (10^5^ spore/mL) and added (100 µL) into an agar plate and dispensed uniformly on the surface of the plate. Small wells were cut in the agar plate using a cork borer (6 mm). A fixed volume of different extracts and amphotericin B (PAA Lab., Germany) as positive control at a concentration of 500 µg/well were loaded in the wells. The plates were incubated at 29 °C for 72 h. The diameter of the inhibition zone around each well was then recorded in four different directions.

### 3.7. Statistical Analysis

The antimicrobial activities, total saponin contents and profiling of phenolic and flavonoid compounds were analyzed using analysis of variance (ANOVA) with Statistical Analysis System (SAS) Version 9 (SAS Institute, Cary, NC, USA). Significant differences among means from triplicate analyses (p < 0.05) were determined by Duncan’s Multiple Range Test.

## 4. Conclusions

This preliminary screening showed interesting results and indicated the antimicrobial potential of the three varieties of *Labisia pumila* Benth. In light of these experiments, it could be concluded that the different fractions of *Labisia pumila* aerial parts exhibited interesting antibacterial and antifungi activities against all strains tested in this study. The antimicrobial activity of *Labisia pumila* could be attributed to various phytochemical constituents (flavonoid, phenolic and saponin compounds) present in the respective crude extracts. The purified components may have even more potency with respect to inhibition of microbes. Further work on the types of phytoconstituents and purification of individual groups of bioactive components may reveal the exact potential of the plant to inhibit several pathogenic microbes and encourage the development a novel broad spectrum herbal antimicrobial formulation in the future.
